# Laser capture microdissection in *Ectocarpus siliculosus*: the pathway to cell-specific transcriptomics in brown algae

**DOI:** 10.3389/fpls.2015.00054

**Published:** 2015-02-10

**Authors:** Denis Saint-Marcoux, Bernard Billoud, Jane A. Langdale, Bénédicte Charrier

**Affiliations:** ^1^Department of Plant Sciences, University of OxfordOxford, UK; ^2^CNRS, Sorbonne Université, UPMC Univ Paris 06, UMR 8227, Integrative Biology of Marine Models, Station Biologique de RoscoffRoscoff, France

**Keywords:** laser capture microdissection, cell-specific transcriptomics, cell differentiation, seaweed, brown algae

## Abstract

Laser capture microdissection (LCM) facilitates the isolation of individual cells from tissue sections, and when combined with RNA amplification techniques, it is an extremely powerful tool for examining genome-wide expression profiles in specific cell-types. LCM has been widely used to address various biological questions in both animal and plant systems, however, no attempt has been made so far to transfer LCM technology to macroalgae. Macroalgae are a collection of widespread eukaryotes living in fresh and marine water. In line with the collective effort to promote molecular investigations of macroalgal biology, here we demonstrate the feasibility of using LCM and cell-specific transcriptomics to study development of the brown alga *Ectocarpus siliculosus*. We describe a workflow comprising cultivation and fixation of algae on glass slides, laser microdissection, and RNA amplification. To illustrate the effectiveness of the procedure, we show qPCR data and metrics obtained from cell-specific transcriptomes generated from both upright and prostrate filaments of *Ectocarpus*.

## Introduction: bringing cell-specific transcriptomics and the development of macroalgae together

Transcriptomics allows the expression profiles of large sets of genes to be monitored in a single experiment, and as a consequence its application has had an impact in virtually every field of biology. Transcriptomics has particularly revolutionized developmental biology because body plans are specified by complex regulatory networks of genes that are expressed in precise spatial and temporal domains. Previously examined with microarrays, transcriptomes are ever more frequently studied using next generation sequencing techniques, an approach termed RNA-Seq (reviewed in Wang et al., 2009). In contrast to microarrays, RNA-Seq is not dependent on the availability of a set of well-defined hybridization fragments, and thus the expression of previously unannotated genes can be monitored. Moreover, RNA-Seq allows for transcriptomic studies to be conducted with organisms for which no genome sequence is available, because transcriptome sequences can be assembled *de novo*. However, this technique has its own pitfalls, requiring intensive bioinformatic analysis of raw sequence reads in order to accurately extract gene expression data.

One of the most powerful but challenging applications of transcriptomics is the analysis of genome wide expression profiles in single cell-types. In some cases this can be achieved by *in vitro* cultivation of a cell type (e.g., Joosen et al., 2007; Wu et al., 2010) but such an approach does not conserve cells in their normal environment and patterns of gene expression are therefore unlikely to match those found *in vivo*. Alternatively, cell-types can be microdissected from whole tissues. Since the mid-1990s, laser capture microdissection (LCM) has been used for this difficult task (Emmert-Buck et al., 1996). Two forms of LCM have been developed (Espina et al., 2006) but the underlying principle in both is that a subset of cells is captured from embedded tissue sections that are visualized using an optical microscope. Current iterations of the technology use slides covered with a plastic membrane onto which sections are attached; a laser cuts out the specific cell-types, which are then propelled or fall by gravity into a collection vessel. In other setups, the laser is used to fuse a thermoplastic component situated above the section to the cell-types of interest, and then the plastic is used to pull the cell-type out of the tissue. For a comprehensive description of the use and applications of this technology see Espina et al. (2006). LCM is the only technique that permits the extraction of deeply embedded cells from within a tissue and the method has numerous advantages compared to other microdissection techniques. For example, compared to techniques that use microcapillaries, it is not limited to surface cells nor does it require the use of fluorescent protein labeling (Karrer et al., 1995; Brandt et al., 1999). However, the technique has several drawbacks, not least that extensive tissue preparation is usually needed before cells can be captured (i.e., fixation, embedding and sectioning). In addition, cell-types are generally identified on the basis of morphological traits which may not be accurately described or easily identified during microdissection, and thus a relatively high operator skill level is required; that said histological staining and/or fluorescent labeling can sometimes be combined with LCM to aid cell-type identification. Finally, because only minute amounts of material are typically collected during laser capture, the amount of nucleic acid that can be extracted is often too low for direct sequencing and thus an amplification step has to be introduced which can lead to non-uniform quantitative changes (Schneider et al., 2004; Boelens et al., 2007; Bhargava et al., 2014). Despite these technical difficulties, LCM has been successfully applied to the study of gene expression in animal cells (Emmert-Buck et al., 1996), plant cells (Nakazono et al., 2003), and most recently fungi (Gomez and Harrison, 2009; Fosu-Nyarko et al., 2010; Teichert et al., 2012). To date, no attempt has been made to use LCM to study gene expression profiles in macroalgae.

Macroalgae are multicellular eukaryotes found in both marine and fresh water. These organisms belong to three phylogenetic branches congruent with the color of their pigments: green and red algae are sub-branches of the Archaeplastida group (or “Plantae”), and brown algae belong to the Stramenopile group (Baldauf, 2008). All three have recently been phylogenetically linked within the SARP megagroup (He et al., 2014). Each macroalgal lineage displays an extreme diversity of body shapes (filamentous, branched, complex three dimensional), sizes (in the range of a few micrometers to several meters high) and life cycles (with or without alternation of generations) (Fritsch, 1945; more recently discussed in Charrier et al., 2012; Leliaert et al., 2012). Such diversity raises important biological questions in relation to development, evolutionary trajectories and adaptation to different environments, and yet macroalgae have received little interest from the scientific community beyond taxonomic descriptions. As a consequence, only a few molecular techniques routinely used in other organisms have been adapted for use with macroalgae. For example, the genomes of only three macroalgae have been sequenced: the brown alga *Ectocarpus siliculosus* (Cock et al., 2010) plus the red algae, *Chondrus crispus* (Collén et al., 2013), and *Pyropia yezoensis* (Nakamura et al., 2013). However, the scientific community is now moving toward molecular studies in macroalgae, notably driven by the potential applications of these organisms for food, biofuel and bioremediation of degraded environments, but equally driven by the potential for understanding the developmental biology of these fascinating organisms.

Here we report the use of LCM to generate cell-specific transcriptomes of the brown alga, *E. siliculosus*. Each technical step and its optimization is described and, as a proof of principle, we present metrics of transcriptomes obtained after isolation of three different cell-types from *E. siliculosus* thallus. Finally, we discuss the potential of this approach to address significant biological questions.

## Growth of *Ectocarpus* on adapted microdissection material

**Figure 1:**
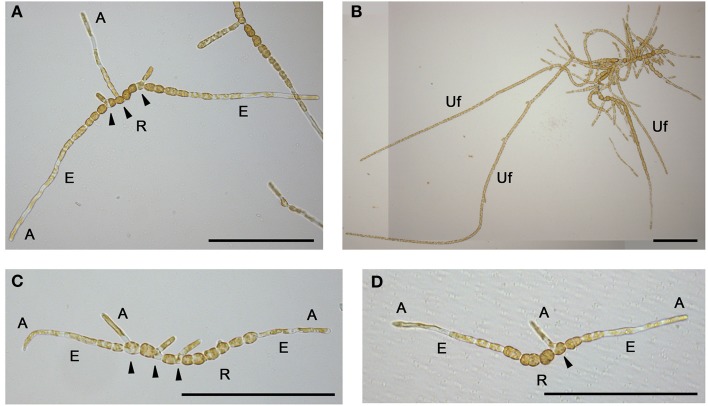
**General morphology and on-slide cultivation of *Ectocarpus* thalli**. Early branching prostrate filament **(A)** and developed thallus showing three upright filaments **(B)**. Morphology of early prostrate filaments on glass **(C)** and PEN membrane **(D)** slides. A, apical; E, elongated; R, round cells; Uf, upright filament. Arrows indicate branching cells within the prostrate filament. Scale bars equal to 200 μm. **(B)** has been reconstructed from two pictures.

Plant and animal tissues are typically prepared for LCM by traditional histological techniques (fix, embed, section and stain) and then sections are mounted on special microscope slides covered with a polyethylene naphthalate (PEN) membrane. Sections are attached to the membrane, and after the laser beam has cut the relevant cell-type, both membrane and cell-type are lifted into the collection vessel. The main purpose of the membrane is to reduce the attraction between section and slide and thus to lower the energy required to lift the sample. Because the early sporophyte of *Ectocarpus* is prostrate and filamentous (Figure 1A), and later stages produce upright filaments that freely float in the growth medium (Figure 1B) (Charrier et al., 2008), we anticipated that fixation and sectioning of material would be problematic. Consequently, we attempted to grow sporophytes directly on PEN membrane slides in order to position the thalli longitudinally. This approach minimized the number of steps required for sample preparation, which in turn minimized the risk of RNA degradation. To inoculate the slides, mito-spores were obtained from mature sporophytes by simulating a low tide as reported in Le Bail and Charrier (2013). Spores were suspended in a droplet of NSWp medium (Starr and Zeikus, 1993) and deposited onto both PEN-coated and uncoated (as a control for normal thallus development) slides. Slides were then placed in petri dishes containing a few NSWp droplets to create a humid environment, and were kept overnight in the dark to allow the spores to settle and fix onto the slide surface. Slides were then fully immersed in NSWp and cultured essentially as described in Le Bail and Charrier (2013) for 2 or 4 weeks to obtain prostrate or upright filaments, respectively. When inoculating the slides, care was taken to ensure that the spore concentration was low enough for filaments to grow without overlapping. In this way, the different cell types within the thallus, elongated (E-type) and round (R-type) (Le Bail et al., 2008a), could be identified by directly examining the slide with a light microscope. *Ectocarpus* thalli grown both on control glass slides (Figure 1C) and PEN membrane slides (Figure 1D) showed the expected morphology after 2 weeks of development, with prostrate filaments starting to produce branches from the center cells as described in Le Bail et al. (2008a). Furthermore, the different cell types were present and arranged in the expected order along the filaments. Older thalli developed upright filaments on both slide types (not shown). We concluded that both overall morphology and cellular differentiation within the thallus were unaffected by growth on PEN membrane. Therefore, cultivating *Ectocarpus* directly on PEN membrane slides appeared suitable preparation for LCM.

## Chemical fixation of *Ectocarpus* filaments

**Figure 2:**
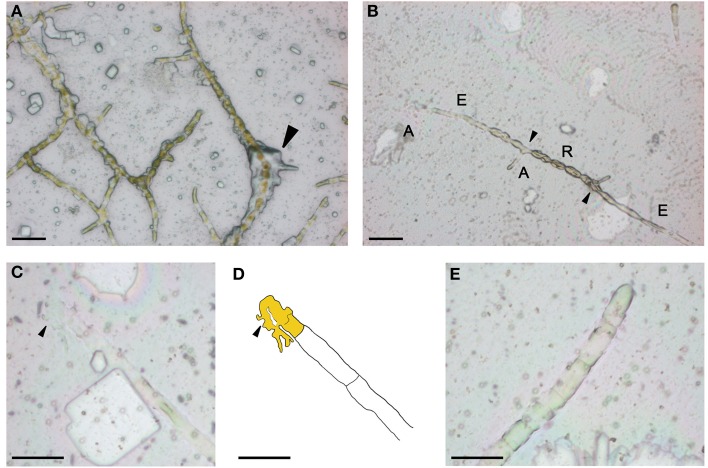
**On slide acetone fixation of *Ectocarpus* filaments**. Filaments were fixed for 30 min in acetone without **(A)** and with **(B)** a prewash in water. Close-up of a burst A-type cell **(C)** and its schematic **(D)**. General view of upright filaments **(E)**. A, A-type; E, E-type; R, R-type. Arrow(s) indicate in **(A)** salt precipitate, in **(B)** branching cells, in **(C)**, and **(D)** discharged cell content. Scale bar equals to 50 μm in **(A,B)**, 25 μm in **(C–E)**. The apical cell is depicted in yellow in **(D)**.

To facilitate handling and preservation of *Ectocarpus* filaments for LCM, chemical fixation was needed. Two major types of fixative are commonly used for histology; precipitative fixatives that coagulate the cell content by essentially denaturing the proteins (e.g., ethanol based), and cross-linking fixatives that create chemical bonds between proteins and lipids (e.g., aldehyde based) (Ruzin, 1999). As compared to precipitative fixatives, cross-linking fixatives have been shown to decrease both quality and quantity of RNA extracted from animal or plant tissues (Goldsworthy et al., 1999; Nakazono et al., 2003) and thus are avoided in LCM-based transcriptomic studies. Acetone is a precipitative fixative that has been successfully used for LCM with different plant species, e.g., maize (Zhang et al., 2007; Brooks et al., 2009), *Arabidopsis* (Gandotra et al., 2013), rice (Takahashi et al., 2010; Ogo et al., 2014), and strawberry (Hollender et al., 2014), as well as *Marchantia polymorpha* and *Physcomitrella patens* (DS-M and JAL, unpublished). Consequently, we attempted to fix slide-grown *Ectocarpus* thalli by directly immersing the slides in a solution of 100% acetone for 30 min. The morphology of the fixed thalli was relatively well conserved but clear identification of cell-types was impaired by a thick precipitate of salts from the culture medium that surrounded the filaments (Figure 2A). Therefore, in order to improve cell-type identification and to remove precipitated salts, we tested a number of fixation procedures (Table 1).

**Table 1:**
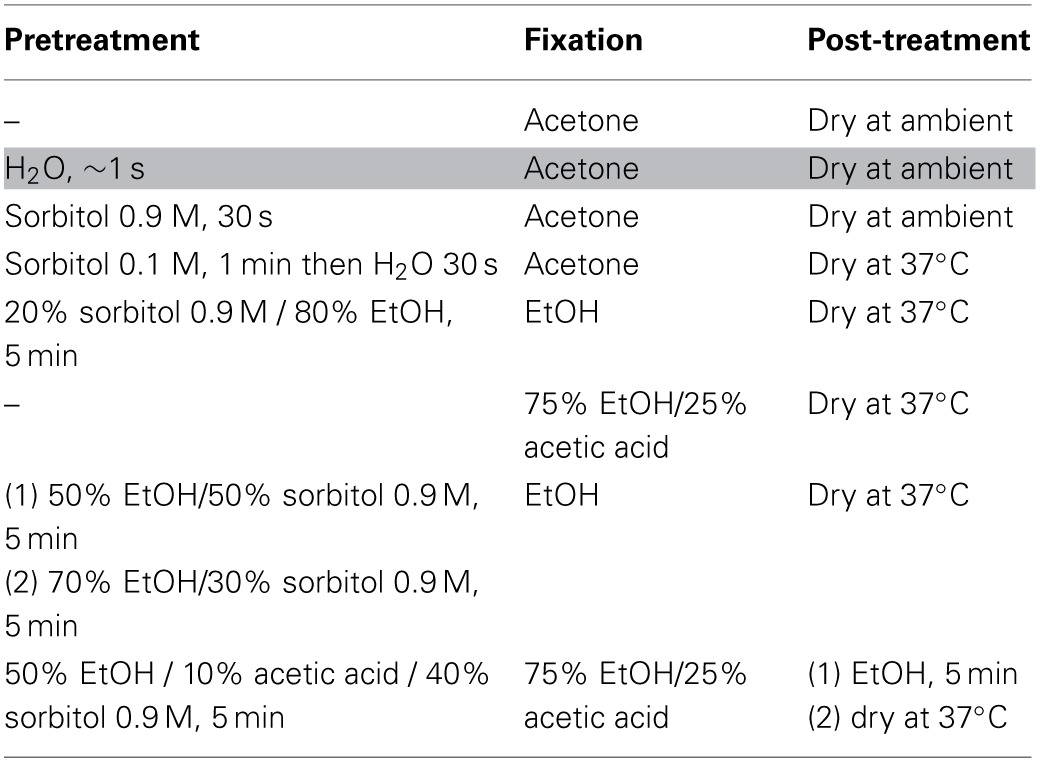
**List of treatments tested to fix *Ectocarpus* filaments for LCM**.

*Fixation and post-treatments were carried out for 30 min unless stated otherwise. Selected procedure is shaded in gray*.

Salt contamination of filaments was greatly reduced when the PEN membrane slide was quickly washed (1 s) in sterile Millipore-filtered water and then the slide edges dried with tissue prior to fixation in 100% acetone. As a consequence, a much clearer view of cell-types along the filaments was obtained (Figure 2B). However, this procedure triggered some morphological changes, particularly of R-type cells which lengthened and became ovoid. Another consequence was that some of the apical cells (A-type) at the tip of the prostrate filaments burst (Figures 2C,D). Because *Ectocarpus* filaments expand by tip growth (Le Bail et al., 2008a), this effect was likely due to a weakness of the cell wall at the tip of these cells. Upright filaments appeared well conserved (Figure 2E) and in contrast to A-type cells, no lysis of the upright filament apical (UA-type) cells was observed.

To further improve the fixation procedure and to reduce morphological changes and the rupture of A-type cells, we tested pretreatments consisting of washes in a water-based solution supplemented with sorbitol. The addition of sorbitol aimed to reduce the osmotic shock provoked by pure water (0.95 M sorbitol is isotonic with marine water). Pretreatments with sorbitol greatly improved morphological preservation and cellular integrity, but unfortunately led to a layer of sorbitol sticking to the surface of the slide, rendering laser cutting and light pulse lifting ineffective. We also attempted to fix *Ectocarpus* in Farmer's fixative (75% EtOH, 25% acetic acid) which is the most commonly used fixative in LCM-based transcriptomic studies (Takahashi et al., 2010 and other numerous publications). However, we observed no improvement over acetone-based fixation and in some cells (mostly E-type) walls were visibly contracted. Therefore, the procedure in which the acetone fixation was preceded by a quick wash in pure water was adopted for all subsequent work. The slight morphological changes and the rupture of some A-type cells did not impair laser capture because the content discharged from A-type cells remained fixed on the slide as shown in Figure 2C. As such, capture of A-type cell contents was still possible, providing the limits of the cell and its content could be recognized.

Fixed slides were stored at room temperature in boxes that had been washed with RNAaseZap (Life Technologies) and that contained silica gel. LCM was performed within a few days after fixation.

## Laser capture of *Ectocarpus* apical and branching cell-types

*Ectocarpus* displays a high level of developmental plasticity, both during the growth of filaments (Nehr et al., 2011) and in the branching process (Le Bail et al., 2008a). Hence, although germinated spores were deposited on slides at the same time, a large spectrum of developmental stages was observed after 2 (or 4) weeks.

**Figure 3:**
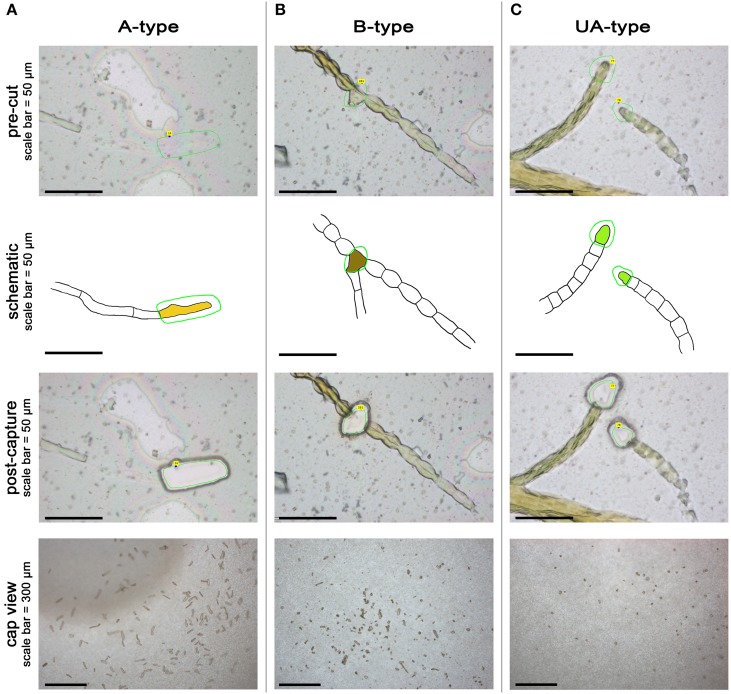
**Laser capture microdissection of *Ectocarpus* cells**. Pre-cut, associated schematic, post-capture and cap view for A-type **(A)**, B-type **(B)**, and UA-type **(C)** cells. Green line represents the laser track in PalmRobo software (pre-cut pictures) and schematics. The A-type cell is represented in yellow in **(A)**, the B-type cell in brown in **(B)**, and the UA-type cell in bright green in **(C)**.

In the first attempt to transfer LCM technology to *Ectocarpus*, we focused on the two types of apical cells present in the sporophyte body: A-type cells from prostrate filaments and UA-type cells from upright filaments (Figures 3A,C). To also test the feasibility of using LCM on less accessible cells, we captured branching (B-type) cells from prostrate filaments (Figure 3B). Cells were carefully selected on the basis of both morphological and positional criteria. Selection of A-type cells was difficult because of their clear content and apparently thin cell wall. Transverse cell walls between A-type cells and the sub-apical cells below them were not always identifiable and in such cases, cells were not selected for capture. Where cells had ruptured, as stated above, careful attention was needed to determine the limits of the cell contents. B-type cells were defined as branching cells that have produced a branch of no more than two cells long, including the new branch apical cell. B-type cells were not selected on their morphology because any cell type, with the exception of A- and UA-types, can produce a bud, although more than 65% of the branches are produced on R-type cells (Le Bail et al., 2008a).

Microdissection was performed using a Carl Zeiss PALM MicroBeam unit at 40x magnification. Laser tracks were drawn with the PalmRobo 4.5 software driving the machine around the cells of interest (see pre-cut and schematic Figure 3). Laser settings were adjusted to ensure the lowest possible radiation energy whilst maintaining effective dissection and light lifting to the collection vessel. Laser power ranged from 40 to 48 depending on the thickness of the cells: typically, laser energy was reduced to capture A-type cells because the cell wall was easy to cut, raised to capture B-type cells that have a much thicker cell wall, and adjusted in between for UA-type cells. Other settings remained constant (laser focus 35; LPC delta +27; LPC focus 35; cutting speed 14; 1 cycle of cutting). The laser spot was thin enough to cut between the cells without damaging the cellular content, and even the isolation of B-type cells was achieved with only minimum damage to the surrounding cells (see post-capture Figure 3B). Dissected cells were propelled into collection vessels (tube caps containing an adhesive polymer) by directing the light focus on the side of the section away from the sample using the RoboLPC function. This approach further minimized potential RNA degradation from laser pressure. However, sometimes it was necessary to use the CenterRoboLPC function, in which the pulse of light was directed toward the center of the section, increasing the potential for RNA degradation. Such treatment was necessary when liquid had infiltrated between the membrane and the glass slide during the prolonged immersion of the slides in NSWp medium. At fixation, salts contained in the liquid precipitated and formed a cement that deposited in patches between the membrane and the slide (white geometrical areas in Figure 2C). When the dissected cells were situated above such a patch, CenterRoboLPC was required to successfully lift the membrane. As morphological preservation was not always homogeneous across the slide, only well preserved cells were harvested. All captures were imaged in the tube cap (cap view, Figure 3) and pictures were assembled in order to count the effective number of captures done for each cell-type (not shown). Three biological replicates were captured for subsequent transcriptomic and qPCR studies: one replicate for prostrate filaments is defined as a batch of captures comprising the A-type and B-type cells from the same slide; one replicate for UA-type cells was collected from two slides, each independent from the prostrate filament slides. Each slide was independently cultured. Captures were done over the course of a few days in no particular order. Table 2 summarizes the number of captures done per cell type for each replicate.

**Table 2:** **Number of captures and final quantity of amplified cDNA per cell type and per biological replicate**.

		**Replicate 1**		**Replicate 2**		**Replicate 3**	
**Cell type**	**n. cells**	**n. captures**	**amp. (μg)**	**n. captures**	**amp. (μg)**	**n. captures**	**amp. (μg)**
A	1	205	5.0	204	6.3	185	5.8
B	1	195	5.2	200	6.5	180	5.1
UA	1	148	4.0	137	4.2	132	4.3

## RNA extraction and amplification from laser captures

RNA extraction and sequencing from laser-captured cells require procedures to be adapted to deal with minute amounts of starting material. We used the Arcturus PicoPure RNA extraction kit, which is commonly used for extracting RNA from a small number of cells. The quantity of RNA obtained after LCM is typically in the order of several picograms to a few nanograms, depending on the amount and type of cells captured. A quantity of 10 pg of total RNA per cell is commonly quoted, (e.g., Espina et al., 2006), and thus at best, about 2 ng of RNA can be obtained from 200 captures of a single cell-type; this is assuming no variation in RNA content between cells of the same cell type and 100% efficiency during experimental procedures—both unrealistic assumptions. Current technologies for RNA-Seq require a few 100 nanograms for sequencing library preparation. For example, the Illumina TruSeq manual states 100 ng as the minimum starting quantity of total RNA. Consequently, RNA extracted from laser-captured cells cannot be used directly for RNA-Seq but instead has to be amplified.

Linear amplification of RNA based on the Eberwine method (Phillips and Eberwine, 1996) has been the most commonly used procedure to amplify small amounts of RNA in recent years. The method relies on polyA-primed retrotranscription of initial RNA, followed by T7 *in vitro* transcription (IVT) after cDNA second strand synthesis. This approach generates antisense RNA because the T7 promoter is linked to the 5′ end of the polyA hybridizing primer. A second round of amplification can be performed to further amplify the initial quantity of RNA. The relative quantities of each RNA species present in the initial extract are reportedly conserved in the final product, however, this method has several drawbacks. First, the products are notably 3′ biased due to the polyA priming, and this can severely impair *de novo* transcriptome assembly due to lack of representation of 5′ sequences. Second, the method cannot capture non-polyadenylated species of RNA, e.g., long and short non-coding RNA, and microRNAs (for review see Jacquier, 2009; Mercer et al., 2009; Ghildiyal and Zamore, 2009; Fatica and Bozzoni, 2014). Finally, even though the method has been developed in various commercial kits, it is labor intensive and prone to experimental errors due to the large number of steps involved.

Other RNA amplification methods have appeared more recently, generally based on strand displacement or PCR. For example, NuGEN have developed a special chemistry based on RNase H degradation of a hybrid RNA-DNA primer and strand displacement—details can be found in Kurn et al. (2005). Retrotranscription is primed by polyA selection and random primers, which in theory should enable the amplification of non-polyadenylated RNA and thus capture much more transcriptomic information than classical priming. Moreover, this approach should reduce the 3′ bias encountered with single polyA priming. The final product of amplification is a single or double stranded cDNA, which allows the use of DNA based procedures for sequencing library construction—in contrast to IVT based kits where antisense RNA is the end product. When three IVT kits were compared with the NuGEN kit, the lowest 3′ bias was confirmed for NuGen (Clément-Ziza et al., 2009). This study also showed better reproducibility between technical replicates with NuGEN technology, as compared to the others. Both of these features were confirmed in our hands with test amplifications and subsequent qPCR analyses (not shown). These results, plus that fact that the NuGEN procedure appeared extremely facile and quick to work with (completion of the whole procedure within a working day whereas IVT based protocols span several days), led us to amplify RNA extracted from *Ectocarpus* laser captures using NuGEN technology.

**Figure 4:**
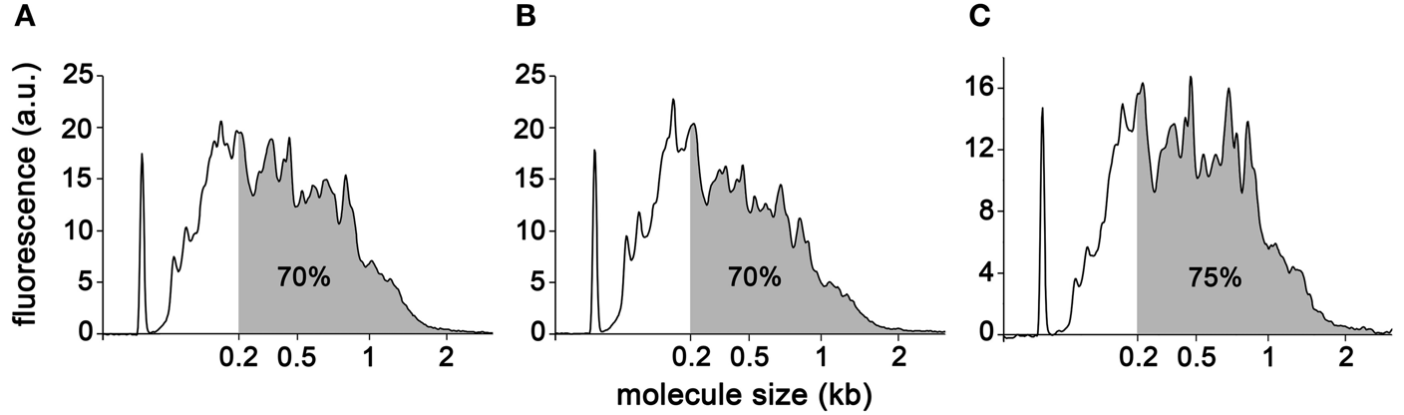
**BioAnalyzer traces of amplified cDNAs**. A-type **(A)**, B-type **(B)**, and UA-type **(C)** cells. Percentage of DNA molecules greater than 200 nucleotides is indicated.

For each sample, RNA obtained using the Arcturus extraction kit was reduced to a volume in which the entire extract could proceed directly to linear amplification with the Ovation RNA-Seq System v2 kit from NuGEN. All experimental procedures were carried out essentially as described in the kit manual except that the final cDNA purification step was carried out using the Qiaquick PCR purification kit from Qiagen. The samples were processed during three different amplification runs: A-type replicate 1 and replicate 2, and B-type replicate 1; A-type replicate 3, and B-type replicate 2 and replicate 3; UA-type replicates altogether. The quantities of amplified cDNA obtained ranged from 4 to 6.5 μg (Table 2), with no obvious correlation between the amount of starting material and the final amount of cDNA (compare A-type cells replicates 1 and 2). Both of these observations are in good agreement with results reported previously (Clément-Ziza et al., 2009), and with results obtained with RNA extracted from other organisms (DS-M and JAL, unpublished). To further characterize the end product of NuGEN amplification, we analyzed the cDNA obtained on a BioAnalyzer with the RNA nano chips. Traces showed amplified product lengths ranging from less than 100 nucleotides to ~2 kb for A-type and B-type replicate 1 and UA-type replicate 1 (Figures 4A–C respectively). By calculating the area under the curve approximated by the trapezoidal rule, we determined that 70–75% of the cDNA species were longer than 200 nucleotides and 37% longer than 500 nucleotides. The very spiky distribution pattern was unexpected, and was in contrast to the smooth curves previously reported (Clément-Ziza et al., 2009). At this stage of the study, we hypothesized that the peaks could be due to abundant species of RNA present in the initial extract—when amplified, these RNAs generated large quantities of identically sized cDNA molecules. Replicates 2 and 3 showed essentially similar cDNA distribution patterns (not shown).

## Analysis of amplified cDNA from the three cell types by qPCR

**Figure 5:**
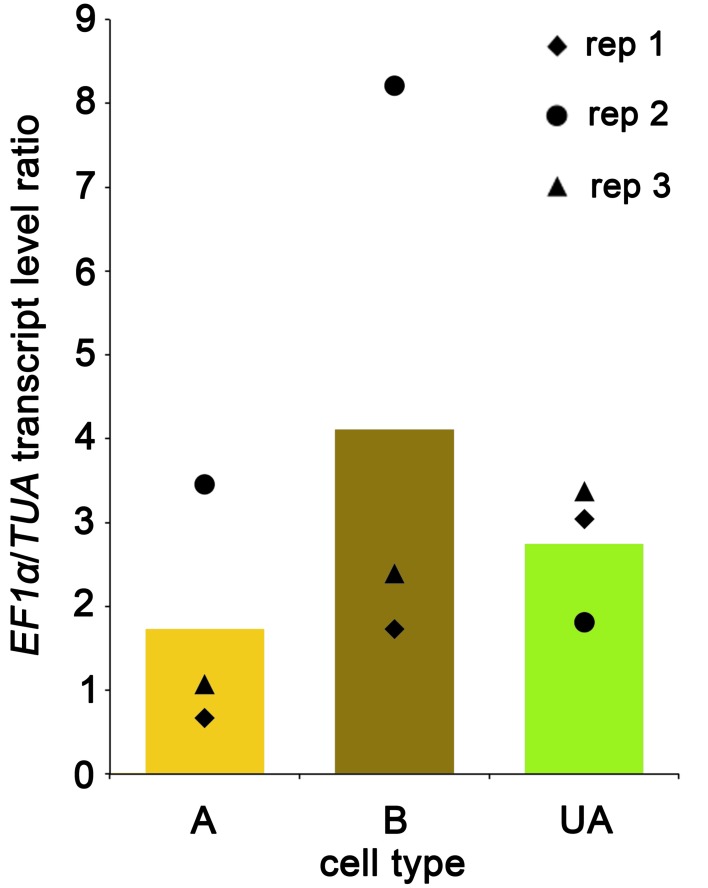
**Ratio of *EF1α* and *TUA* transcript levels in amplified cDNA samples from LCM derived A-type, B-type and UA-type cells**. *EF1α*/*TUA* ratio is shown in the three biological replicates. Average expression by cell type is depicted as a histogram.

To test the amplified cDNAs for fidelity of representation of *Ectocarpus* transcriptomes, we measured transcript levels of two “housekeeping” genes: *Elongation Factor 1α* (*EF1α*) and *Tubulin subunit α* (*TUA*). These genes were shown to be expressed at a high level: ~2500 transcript copies present per ng of total RNA for *EF1α* and ~1200 for *TUA* (Le Bail et al., 2008b). Gene transcripts were quantified by qPCR, with oligonucleotides located in the 3′-UTR region of each gene, in the three biological replicates of A-type, B-type and UA-type cells. Figure 5 shows that while replicate 1 and 3 displayed similar ratios of *EF1α*/*TUA* transcript levels in the three cell types, replicate 2 was more heterogeneous, with high *EF1α*/*TUA* ratios in A-type and B-type cells and a lower ratio in UA-type cells. Nevertheless, the *EF1α*/*TUA* ratios of the nine samples (ranging from 0.7 to 8.2, Supplementary Material) were reasonably close to the ratios reported for these genes in overall *Ectocarpus* tissues (1.15–2.95; Le Bail et al., 2008b). This shows that genes with relatively high expression levels can be amplified and their transcript level quantified with reasonable accuracy, provided that the number of biological replicates is adequate to compensate for any possible bias introduced by the RNA amplification process. Transcript levels of the Ubiquitin-Conjugating Enzyme gene (*UBCE*) were also measured in the amplified cDNAs. Although *UBCE* transcripts could be amplified in these samples, reliable quantification was not possible because transcript levels were too variable between the three replicates (*EF1α*/*UBCE* ratios ranging from ~8 to ~300, not shown). Notably accumulation levels of *UBCE* transcripts are much lower than *EF1α* and *TUA* transcripts, as reported in Le Bail et al. (2008b) (~60 transcript copies per ng of total RNA). As such it can be deduced that quantitative biases were generated during NuGEN RNA amplification of lowly expressed genes, while amplification of moderately to highly expressed genes was reasonably accurate.

## Metrics of A-type and B-type cell transcriptomes

Amplified A-type and B-type cDNAs were paired-end sequenced on an Illumina HiSeq 2000 sequencer at the Beijing Genome Institute using a library with an insert size of 170 bp. This insert size ensured that the majority of molecules present in amplified cDNA (>70%) were represented. Reads were cleaned for low quality and adapter sequences, and approximately 13 million clean reads were obtained for each sample. Every read had an overall Q20 > 97% (base call quality above 20).

We first examined the ribosomal RNA (rRNA) contamination in reads using SortMeRNA v1.7 (Kopylova et al., 2012) with default parameters and a database containing all rRNA species provided with the software. Somewhat surprisingly, reads contained rRNA contamination of up to 94.37% (Table 3). Contamination from organellar rRNA accounted for approximately 70% of the total whereas nuclear encoded rRNAs accounted for about 20%. rRNA contamination is clearly possible given that first strand priming in NuGEN technology is dependent both on polyA and random priming. We also observed a high degree of rRNA contamination (50% on average) on reads generated from other photosynthetic organisms (DS-M and JAL, unpublished). Moreover, a recent study reported 25% contamination from mitochondrial rRNAs in reads generated using NuGEN amplified RNA from human cells (Adiconis et al., 2013). However, these results are in strong disagreement with other reports where only a very small proportion (0.5–3.5%) of the reads were found to be of ribosomal origin (Tariq et al., 2011; Sun et al., 2013). Notably, the spiky BioAnalyzer profiles observed for our cDNA samples (Figure 4) are likely due to this rRNA contamination. It is possible that RNA extraction efficiency and the amount of initial template RNA are two parameters that determine the final degree of rRNA contamination in amplified cDNA. However, we have noted significant rRNA contamination (~50%) regardless of species, sample type (LCM or whole tissue) or initial amount of template RNA (DS-M, and JAL, unpublished).

**Table 3:**
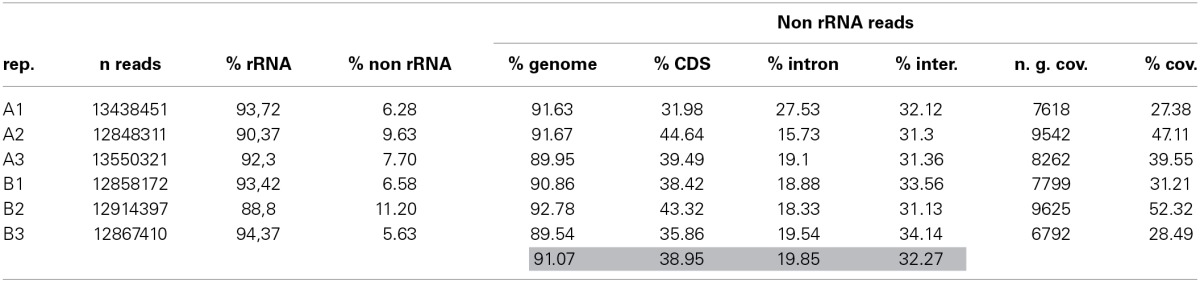
**Metrics determined for the A-type and B-type triplicates**.

*rep. stands for replicate, inter. for intergenic, n. g. cov. for number of gene covered and cov. for coverage; averages shaded in gray*.

After filtering out rRNA reads from the different replicates, we examined the overall characteristics of the reads using FastQC (www.bioinformatics.babraham.ac.uk/projects/fastqc/). We noted the existence of highly repeated kmers at the beginning of reads. The kmers assembled as a 14 nucleotide long sequence (AACTTTGTGTTTGA) that was present at the beginning of ~0.3% of reads. Truncations of the sequence toward the 3′ end were also detected at high rates explaining the highly repeated kmers revealed by FastQC. When attempting to further extend the sequence, a 33 nucleotide long sequence was extracted that forms a hairpin with a loop of 5 nucleotides in the middle (TCAAACACAAAGTTACCTAAACTTTGTGTTTGA). This long sequence was associated with random reads, at no precise location along the reads, and was present in about 0.1–1.5% of the reads either before or after rRNA filtering. Finding this hairpin prompted us to check our other NuGEN amplified transcriptomes—both the hairpin and its 3′ moiety were identified (DS-M and JAL, unpublished). Similar sequences could not be identified in any of our other RNA-Seq data that was generated on the same Illumina platform but from non-amplified total RNA. Given the non- random location of the 3′ stem of the hairpin, i.e., at the beginning of the reads, and the specificity of the hairpin to the NuGEN amplified cDNA, we believe this sequence results from the amplification process. This result is in accordance with a previous study that hypothesized the formation of hairpins during NuGEN amplification based upon biochemical evidence (Head et al., 2011). We could identify the 5′ stem of the hairpin at the end of a few reads but not at a frequency suggesting that an obvious bias was introduced by the technology. Before further characterization of the reads, we removed the hairpin and its 3′ moiety by trimming the 5′ ends using a customized script.

Reads were finally trimmed using sickle (Joshi and Fass, 2011), and then the resulting read pairs and single reads were mapped onto the *E. siliculosus* genome and transcriptome (Cock et al., 2010) using Bowtie (Langmead et al., 2009) and TopHat (Trapnell et al., 2009; Kim et al., 2013). Reads mapped at a rate of 91.07% on average against the genome (Table 3), a value much higher than the 60 and 75% mapping values reported elsewhere (Tariq et al., 2011; Sun et al., 2013). More specifically, reads mapped at an average rate of 38.95, 19.85, and 32.27% against transcripts, introns, and intergenic regions respectively (Table 3). Some variability was detected (for example between A-type replicates 1 and 2) in terms of differential mapping between transcripts and introns, but mapping in intergenic regions was fairly homogenous. These values compared well with those published in Tariq et al. (2011) and Adiconis et al. (2013), although both studies reported inverted mapping values between intron and intergenic regions. The mapping rate to transcripts was also in the range of that found in Sun et al. (2013).

**Figure 6:**
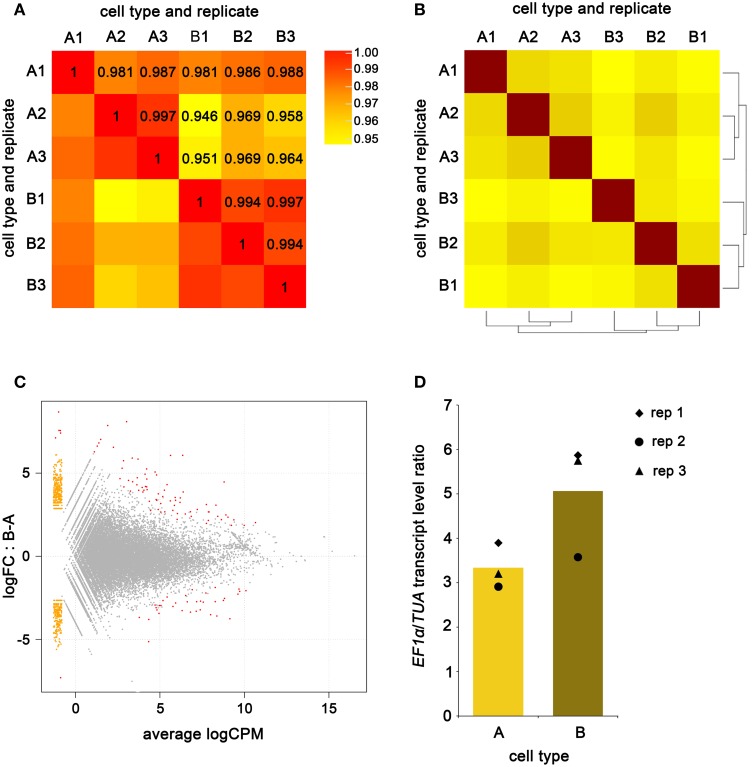
**Analysis of A-type and B-type cell transcriptomes**. Pairwise read count Pearson's correlation between A-type and B-Type cells **(A)**. Sample clustering of the three biological replicates of A-type and B-type cell transcriptomes **(B)**. MA-plot showing the differential expressed genes (red dots), non-differentially expressed genes (gray dots) and genes for which one cell type has a read count equal to zero (orange dots) between A-type and B-type cells **(C)**; logFC stands for log of fold-change and logCPM stands for log of count per million. Transcript level ratio of *EF1α*/*TUA* in the three biological replicates of A-type and B-type transcriptomes **(D)**; average expression by cell type is depicted as a histogram.

Reads from A-type and B-type cells mapped to 10613 (64%) and 10583 (63.8%) transcripts respectively over the 16580 transcripts defined in the *Ectocarpus* transcriptome. The number of transcripts covered by the reads from each replicate and cell type is significantly variable, and the average coverage along these transcripts is relatively low (from 27 to 53%) (Table 3). The variation is likely a consequence of lowly expressed genes being captured by just one or a few reads, and in one replicate only. The two cell types had 9486 transcripts in common and in combination spanned a total of 11710 transcripts (70.6%). The average read count per transcript was 30.9 and 29.6 for A-type and B-type cells respectively. The average Pearson correlation computed from read counts between replicates of the same cell type was at least 97% (Figure 6A), a value slightly above the correlations found in previous reports using NuGEN technology (Clément-Ziza et al., 2009; Tariq et al., 2011; Adiconis et al., 2013). As expected, within cell-type replicates correlated better than between cell type comparisons with the exception of A-type replicate 1, which showed a similar correlation with other A-type and with B-type transcriptomes (Figure 6A). Sample clustering analysis carried out using the DESeq R package (Anders and Huber, 2010) showed that within cell-type replicates clustered together (Figure 6B), thus confirming the Pearson correlations.

Differential gene expression between the A- and B-type cells was analyzed using the EdgeR package (Robinson et al., 2010) with a maximum False Discovery Rate of 10^−5^ and represented as a MA-plot (Figure 6C). A total of 114 transcripts were differentially expressed: 36 transcripts were detected at higher levels (fold-change of 3.7–293) in A-type cells than B-type cells, and 78 transcripts were detected at higher levels (fold-change of 3.6–764) in B-type cells than A-type cells. *EF1α* and *TUA* transcript levels were quantified in A-type and B-type cells by counting the number of reads that matched (with at most 1 substitution) to the respective mRNA sequence, and then calculating the ratio between the two (Figure 6D and see Supplementary Material). The two genes showed expression ratios close to the values reported in qPCR. Variations between the biological replicates were less obvious in the RNA-Seq data than in the qPCR data. As in qPCR experiments, *UBCE* read count was low, and variable within the same cell-type (not shown). These findings confirmed that accurate quantification of lowly expressed genes is difficult to achieve after RNA-Seq of amplified cDNAs.

## General conclusion

We have shown here that LCM can be used to isolate specific cell-types from filaments of the brown alga *E. siliculosus*. We have established a slide culture and chemical fixation procedure that permits easy cell identification and capture, and have developed an RNA extraction and amplification method that facilitates cell-type specific transcriptomics studies. As demonstrated by qPCR and RNA-Seq, amplified cDNAs were suitable material for quantification of cell-type specific gene expression profiles.

Bioinformatic analysis showed that the proportion of non rRNA reads in our sequences was low, however, the quality of those reads was high with over 90% aligning to the *Ectocarpus* genome. At least a third of the reads mapped to annotated coding sequences, a proportion that was sufficient to identify 114 differentially expressed genes between A-type and B-type cells of the prostrate filaments. Overall, 11710 transcripts were detected in the two cell types, representing 70% of the expected *Ectocarpus* transcriptome. Moreover, another third of the reads mapped onto intergenic regions, a result that could reflect pervasive transcription (Clark et al., 2011). Analysis of those reads will likely unravel interesting features of this yet mysterious characteristic of eukaryotic genomes.

Although NuGEN technology allows for the detection of non-polyadenylated transcripts such as non-coding RNAs, it is not well suited for generating LCM derived transcriptomes in *Ectocarpus*. The small number of non rRNA reads obtained prevents reliable quantification of lowly expressed transcripts, as exemplified by the variable quantification of *UBCE* transcript by qPCR and RNA-Seq. Some further optimization is therefore needed at this step. For example, rRNA depletion could be implemented prior to amplification or more conventional amplification technology like IVT could be used.

*Ectocarpus* is morphologically simple relative to other macroalgae but interesting questions arise from the patterns of cellular differentiation observed in both prostrate and upright filaments (Le Bail et al., 2008a). Cell-specific transcriptomics will add valuable knowledge to our understanding of the developmental processes that underpin bodyplan specification in *Ectocarpus*, and in combination with mutant characterization by cutting-edge techniques such as shore-map (see Billoud et al., 2015) will provide a mechanistic understanding of *Ectocarpus* development.

## Method details

Mito-spores were obtained from *E. siliculosus* strain Ec32 (accession CCAP 1310/4) routinely cultured in a growth chamber in natural sea water supplemented with Provasoli medium (NSWp) (Starr and Zeikus, 1993, as described in Le Bail and Charrier, 2013) with the exception that light conditions were set to a 12 h light:12 h dark cycle. Mito-spores were cultivated on glass slides in the same conditions. Nuclease-free 1.0 polyethylene naphthalate (PEN) membrane slides were obtained from Carl Zeiss Microscopy (#415190-9081-000). Laser capture was performed using a Carl Zeiss PALM MicroBeam unit equipped with an AxioVert 200 microscope and a CryLas UV laser, and assisted by the PalmRobo 4.5 software. Captures were collected in 500 μL tubes with adhesive tube caps from Carl Zeiss Microscopy (#415190-9201-000). The PicoPure RNA extraction kit from Life Technologies (#KIT0204) was used to extract RNA. 10 μL of extraction buffer were added to the polymer tube cap before incubating the tube inverted at 42°C for 30 min. Tubes were then centrifuged at 800 × g for 2 min to collect the extract in the bottom of the tube, before being stored at −80°C until further processing. Frozen tubes were processed according to manufacturer's recommendations. On column DNA digestion was carried out using Qiagen RNase-free DNase I set (#79254). RNA was eluted with 11 μL elution buffer and then vacuum concentrated for 10 min in a SpeedVac (Savant) until the volume decreased to 5 μL. The entire extract was then amplified using the Ovation RNA-Seq System v2 kit from NuGEN (#7102-32) following the manufacturer's protocol. cDNA cleanup was achieved using the Qiaquick PCR purification kit from Qiagen (#28104), cDNAs were eluted in 35 μL 10 mM Tris-HCl. cDNA quantity was determined using a NanoDrop ND-1000 spectrophotometer. cDNA quality was analyzed on a 2100 BioAnalyzer (Agilent Technologies) using RNA nano chips (5067-1511, Agilent Technologies) following recommendations in the NuGEN kit. qPCR was carried out as in Le Bail et al. (2008b). *EF1α, TUA*, and *UBCE* genes were amplified according to Le Bail et al. (2008b) except that 1.5 ng of cDNA were used in a 20 μL reaction volume.

### Conflict of interest statement

The authors declare that the research was conducted in the absence of any commercial or financial relationships that could be construed as a potential conflict of interest.

## Supplementary material

Data Sheet 1 ***EF1α*/*TUA* ratios in qPCR and RNA-Seq**.
